# CSF levels of the BACE1 substrate NRG1 correlate with cognition in Alzheimer’s disease

**DOI:** 10.1186/s13195-020-00655-w

**Published:** 2020-07-20

**Authors:** François Mouton-Liger, Julien Dumurgier, Emmanuel Cognat, Claire Hourregue, Henrik Zetterberg, Hugo Vanderstichele, Eugeen Vanmechelen, Elodie Bouaziz-Amar, Kaj Blennow, Jacques Hugon, Claire Paquet

**Affiliations:** 1 Inserm U 1144, University de Paris, Paris, France; 2 Université de Paris, Paris, France; 3 Center of Cognitive Neurology, Lariboisière Fernand-Widal Hospital, APHP, 200 rue du Faubourg Saint Denis, 75010 Paris, France; 4 Clinical Neurochemistry Laboratory, Sahlgrenska University Hospital, Mölndal, Sweden; 5 Department of Psychiatry and Neurochemistry, Institute of Neuroscience and Physiology, The Sahlgrenska Academy at the University of Gothenburg, Mölndal, Sweden; 6 UK Dementia Research Institute at UCL, London, UK; 7 Department of Neurodegenerative Disease, UCL Institute of Neurology, Queen Square, London, UK; 8 ADX Neurosciences, Ghent, Belgium; 9 Department of Biochemistry, Lariboisière Fernand-Widal Hospital, APHP, Paris, France

**Keywords:** NRG1, Aβ, BACE1, CSF, Biomarkers, Alzheimer’s disease, Cognition

## Abstract

**Background:**

The presynaptic protein neuregulin1 (NRG1) is cleaved by beta-site APP cleaving enzyme 1 (BACE1) in a similar way as amyloid precursor protein (APP) NRG1 can activate post-synaptic receptor tyrosine-protein kinase erbB4 (ErbB4) and was linked to schizophrenia. The NRG1/ErbB4 complex is neuroprotective, can trigger synaptogenesis and plasticity, increases the expression of NMDA and GABA receptors, and can induce neuroinflammation. This complex can reduce memory formation. In Alzheimer’s disease (AD) brains, NRG1 accumulates in neuritic plaques. It is difficult to determine if NRG1 has beneficial and/or detrimental effects in AD. BACE1 levels are increased in AD brains and cerebrospinal fluid (CSF) and may lead to enhanced NRG1 secretion, but no study has assessed CSF NRG1 levels in AD and mild cognitive impairment (MCI) patients.

**Methods:**

This retrospective study included 162 patients suffering from AD dementia (54), MCI with progression to AD dementia (MCI-AD) (27), non-AD MCI (30), non-AD dementias (30), and neurological controls (27). All patients had neurological examinations, brain MRI, and neuropsychological evaluations. After written informed consent and using enzyme-linked immunosorbent assays (ELISAs), CSF samples were evaluated for Aβ1–42, Aβ1–40, total tau (T-tau), phosphorylated tau on threonine 181 (P-tau), BACE1, growth-associated protein 43 (GAP 43), neurogranin (Ng), and NRG1.

**Results:**

Levels of NRG1 were significantly increased in the CSF of AD (+ 36%) and MCI-AD (+ 28%) patients compared to neurological controls and also non-AD MCI and non-AD dementias. In addition, in AD and MCI-AD patients, NRG1 levels positively correlated with Aβ1–42 but not with T-tau, P-tau, and BACE1 levels and negatively correlated with MMSE scores. A longitudinal follow-up study of AD patients revealed a trend (*p* = 0.08) between CSF NRG1 levels and cognitive decline. In the overall population, NRG1 correlated with MMSE and the synaptic biomarkers GAP 43 and neurogranin.

**Conclusions:**

Our results showed that CSF NRG1 levels are increased in AD and MCI-AD as compared to controls and other dementias. CSF NRG1 levels are associated with cognitive evolution, and a major outcome of our findings is that synaptic NRG1 could be involved in the pathophysiology of AD. Modulating brain NRG1 activity may represent a new therapeutic target in AD.

## Background

Alzheimer’s disease (AD) is clinically characterized by cognitive decline including memory disturbances followed by aphasia apraxia and agnosia [1]. Neuropathological lesions include amyloid plaques, neurofibrillary tangles made of hyperphosphorylated tau protein, and synaptic and neuronal degeneration [2]. These brain lesions likely occur one or two decades before the onset of clinical signs [3]. According to the amyloid cascade hypothesis, amyloid precursor protein (APP) is initially cleaved by the secretase: beta-site amyloid precursor protein cleaving enzyme 1 (BACE1) followed by γ-secretase leading to the release of Aβ monomers and oligomers, responsible for synaptic and neuronal degradations [4]. A reduction of cerebrospinal fluid (CSF) Aβ levels or an accumulation of amyloid plaques assessed by positron emission tomography (PET) imaging was detected in cognitively normal individuals [5, 6]. It was demonstrated that brain BACE1 concentrations are enhanced in AD and thus possible that BACE1 levels and activity could be increased early during the clinically silent course of AD evolution [7]. Interestingly, BACE1 has several other substrates at the synaptic level including the protein neuregulin1 (NRG1) [8].

There are four members of the neuregulin (*NRG*) gene in mammals from NRG1 to NRG4. The *NRG1* gene is a very large gene in humans, and there are six isoforms with various N-terminal regions (type I to type VI), all characterized by the presence of an epidermal growth factor (EGF)-like domain [8–12]. These isoforms are found in various mammalian organs and are present in the human brain. The type III NRG1 isoform is the most abundant in humans. NRG1 is a trophic factor that can activate the ErbB receptor (EGF receptor) tyrosine kinases. NRG1 can be cleaved and activated by several proteases including BACE1, γ-secretase, a disintegrin and metalloproteinase (ADAM) 10, and ADAM 17. The BACE1-dependent NRG1/receptor tyrosine-protein kinase erbB-4 (ErbB4) signaling is implicated in many neurobiological processes including development, synaptic plasticity, neuronal survival, and memory modulation. In addition, *NRG1* gene is a candidate gene for schizophrenia [13, 14]. Human genetic studies have shown that variants of the *NRG1* gene increase the risk of psychiatric diseases including schizophrenia, but results are still debated.

In AD, previous experimental work revealed that the effects of NRG1 on cognition depend upon its concentration and the dose-response shows an inverted U curve [9]. It is therefore difficult to determine if NRG1 has beneficial and/or detrimental effects on cognition in AD. It has been shown that BACE1 levels are increased in AD brains (7) and that BACE1 accumulates in the neuritic plaques (15). A previous study has revealed enhanced CSF levels of NRG1 in 10 AD patients [15]. No extensive study has assessed and compared CSF NRG1 and BACE1 levels in AD and MCI-AD patients.

The goal of this study was to assess CSF NRG1 levels and BACE1 concentrations in AD, MCI-AD, non-AD MCI, other dementias, and neurological controls and to compare these results with usual CSF AD CSF and synaptic biomarkers and cognition in the same groups.

## Methods

### Population study

**Fig. 1:**
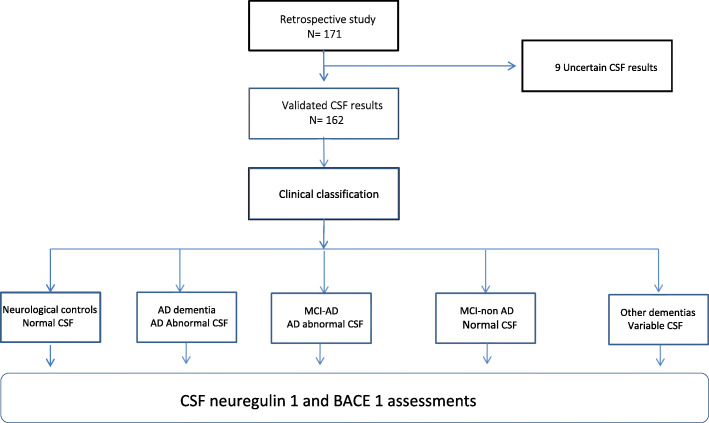
The flow chart used for retrospective inclusion of patients in the protocol

This study is a retrospective cross-sectional monocentric assessment performed at the Cognitive Neurology Center (CNC) in Paris from 2014 to 2016. All individuals included in the study underwent CSF biomarkers analysis for neurocognitive exploration. Change in cognition was evaluated up by the Mini-Mental State Examination (MMSE) score, assessed every 6 months, with a median duration of follow-up of 30 months. Consensus diagnoses were made by several neurologists, geriatricians, neuropsychologists, and biologist experts in CSF biomarkers working at the CNC and in agreement with clinical diagnostic criteria. In the second step, three neurologists (CP, EC, JH) confirmed the diagnosis prior to the sample’s selection. These criteria were used to classify 171 individuals. Nine uncertain (only high total tau (T-tau) or high phosphorylated tau on threonine 181 (P-tau) levels) CSF results were not included, leaving 162 patients who were retained and are shown in the flow chart (Fig. 1) comprising AD (*n* = 56); MCI-AD (*n* = 27); non-AD MCI (*n* = 32); other dementias (*n* = 30) including Lewy body dementia (LBD, *n* = 15), frontotemporal dementia (FTD, *n* = 10), and vascular dementia (VD, *n* = 5); and neurological controls (*n* = 27). Neurological controls included patients with anxiety, depression, or sleep apnea syndrome. The following cutoffs were used to classify AD CSF profiles: Aβ1–42 < 750 pg/mL, ratio Aβ40/42 < 0.055, T-tau > 300 pg/mL, and P-tau > 60 pg/mL. Only patients with 3 abnormal biomarker levels were considered as AD or MCI-AD. Controls had normal levels of CSF tau and CSF P-tau and either normal level of CSF Aβ1–42 or decreased level of CSF Aβ1–42 but normal amyloid ratio Aβ40/42 [16]. In order to assess synaptic alteration, two synaptic markers (GAP 43 and neurogranin (Ng)) were blindly assessed and not used for diagnosis. All patients signed an informed consent, and this study was approved by the Bichat Hospital Ethics Committee of Paris Diderot University.

### CSF sampling

The second and third milliliters of CSF were used and centrifuged to prevent blood contamination. The supernatant was stored at − 80 °C. All analyses of CSF core AD biomarkers (Aβ1–42, Aβ1–40, T-tau, and P-tau) were locally performed in the Department of Biochemistry at Lariboisiere Hospital Paris using commercially available kits from Fujirebio Inc. (INNOTEST®).

### ELISA immunoassay

NRG1 levels were measured in CSF samples using Human NRG1 DuoSet ELISA kit (R&D Systems, Minneapolis, MN), according to the manufacturer’s instructions. Absorbance was read at 450 nm, with a VICTOR plate reader (PerkinElmer, Wellesley, MA), and NRG1 levels were calculated from a standard curve. All analyses were performed at the Inserm Unit 1144 in Paris. CSF Ng analyses were carried out using an ELISA employing the monoclonal antibody NG36 (in-house developed, Mölndal, Sweden), CSF GAP 43 was measured using an ELISA employing the NM4 monoclonal antibody (Fujirebio, Ghent, Belgium), and CSF BACE1 concentration was measured using an ADX 401 and ADX 402 antibody-based ELISA (ADX Neurosciences, Ghent, Belgium) at the Clinical Neurochemistry Laboratory at the Sahlgrenska University Hospital (Mölndal, Sweden), as previously described in detail [17].

### Statistical analysis

Participant characteristics were examined in 5 groups: AD, MCI-AD, non-ADMCI, other dementias, and neurological controls. Proportions were calculated for categorical variables, while means and standard deviations were computed for continuous variables. Comparisons between the groups were assessed using Pearson’s *χ*^2^ or Fisher’s exact test for categorical variables and one-way analysis of variance (ANOVA) for continuous variables. The relationship between CSF NRG1 and group of patients was determined using a linear regression analysis, first unadjusted and secondly adjusted for age and sex, considering the neurological control group as reference. In the third model, we further adjusted on MMSE and CSF Aβ1–42 levels, which were both associated to CSF NRG1 levels in univariate analyses. We first analyzed the group of AD and MCI-AD patients independently and then combined these two groups of patients in one category. CSF NRG1 and CSF BACE1 levels were log-transformed in linear regression analyses to improve the normality of their distribution. The ability of CSF NRG1 and CSF BACE1 to discriminate AD patients versus controls was evaluated using ROC curves analysis, and we established optimum cutoffs that maximized the Youden index. We then studied the association between CSF NRG1 and other characteristics and CSF biomarkers in AD and (AD+MCI-AD) groups of patients using Spearman correlation coefficients analysis and plotted the MMSE score in the function of CSF NRG1 levels. Finally, we determined the association between CSF NRG1 and longitudinal decline of MMSE (defined as last follow-up MMSE minus initial MMSE divided by the time in months between the 2 measurements) by using correlation coefficients analysis and plotted this association. We applied a Bonferroni correction to take into account the multiple comparisons between all the biomarkers in the correlation analysis and the risk of false-positive findings and considered *p* < 0.005 as statistically significant in these analyses.

Statistical analyses were performed using SAS version 9.4 (SAS Institute, Cary, NC, USA).

## Results

### Flow chart

All 162 included patients were retrospectively selected according to the flow chart depicted in Fig. 1.

### Characteristics of the patients and CSF AD biomarkers

The characteristics of the patients are shown in Table 1. AD and MCI-AD patients were slightly older than the other groups, and 55.6% of individuals were women. MMSE scores were significantly lower in AD and other dementia groups. The number of *APOE* ε4 carriers was significantly increased in AD patients. Aβ1–42 CSF levels were significantly reduced in AD and MCI-AD, and T-tau and P-tau were increased in these groups.

**Table 1:** Characteristics of the population study

Characteristics	Overall (*N* = 162)	Controls (*N* = 27)	AD (*N* = 54)	MCI-AD (*N* = 20)	Other MCI (*N* = 31)	Other dementias (*N* = 30)	*p* value
Age, years, mean (SD)	66.6 (9.5)	62.0 (11.3)	69.4 (7.9)	70.2 (8.0)	61.5 (9.6)	68.7 (7.6)	< 0.001
Women, *n* (%)	90 (55.6)	23 (85.2)	33 (61.1)	12 (60.0)	11 (35.5)	11 (36.7)	< 0.001
MMSE, mean, (SD)	23.4 (4.9)	26.0 (3.3)	20.3 (4.7)	27.0 (1.7)	25.8 (2.8)	22.0 (5.4)	< 0.001
Baccalaureate degree or higher, *n* (%)	64 (44.8)	13 (56.5)	18 (37.5)	12 (66.7)	11 (37.9)	10 (40.0)	0.05
APOE ε4carriers, *n* (%)^a^	65 (43.9)	4 (16.0)	33 (64.7)	7 (38.9)	8 (30.8)	13 (46.4)	< 0.001
CSF biomarkers, pg/mL, mean (SD)							
CSF Aβ1–42	751.1 (290.8)	951.9 (285.6)	548.3 (153.6)	695.5 (270.6)	930.0 (287.3)	787.5 (261.1)	< 0.001
CSF Aβ1–40	11,920 (5613)	10,698 (5078)	14,276 (5846)	14,223 (7197)	10,788 (3624)	8202 (3122)	< 0.001
CSF Aβ42/40 ratio	0.076 (0.042)	0.10 (0.047)	0.043 (0.015)	0.053 (0.012)	0.094 (0.031)	0.11 (0.043)	< 0.001
CSF tau	400.0 (272.7)	192.5 (76.3)	664.2 (264.8)	478.5 (139.3)	208.2 (69.7)	256.9 (164.7)	< 0.001
CSF P-tau	60.7 (32.0)	34.7 (11.1)	92.4 (27.5)	76.3 (14.5)	38.5 (10.8)	39.8 (17.4)	< 0.001
CSF BACE1	116.9 (42.0)	99.3 (30.4)	140.0 (46.0)	134.6 (38.3)	106.4 (29.6)	89.3 (28.8)	< 0.001
CSF NRG1	320.1 (134.2)	267.7 (104.2)	364.7 (149.2)	342.6 (161.5)	304.9 (113.0)	287.5 (106.5)	0.01

^a^APOE was missing for *n* = 14 participants

### CSF NRG1 and BACE1 results

**Fig. 2:**
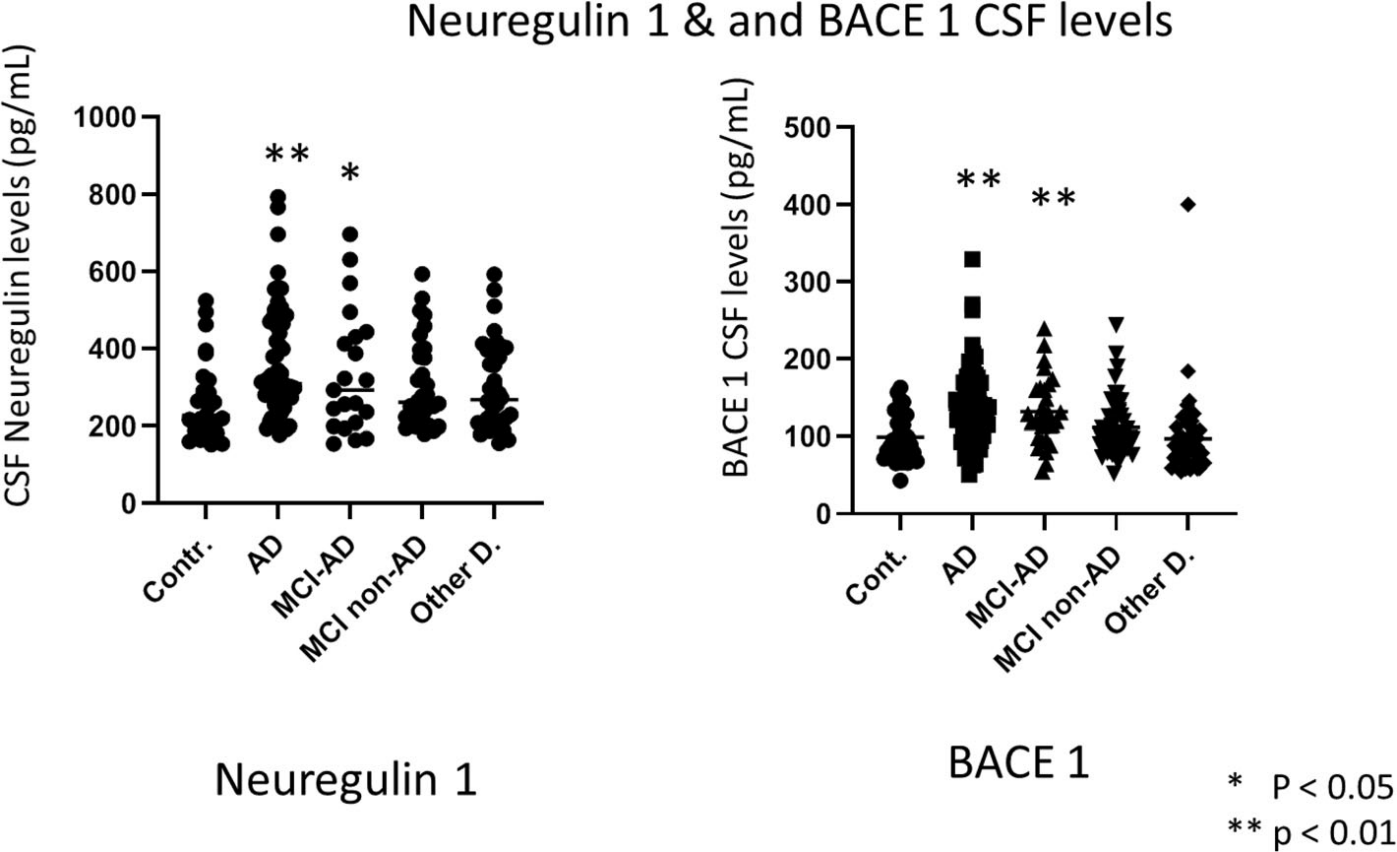
The distribution of CSF NRG1 and BACE1 levels in various groups of patients comprising AD, MCI-AD non-AD MCI, other dementias, and neurological controls. Two-sided ANOVA tests *p < 0.05, **p < 0.01

**Fig. 3:**
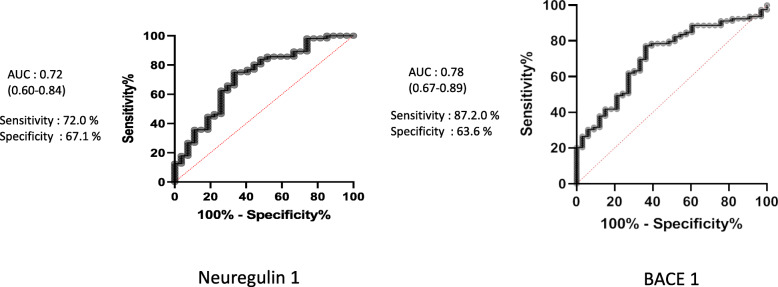
The discriminatory power curve of CSF NRG1 and BACE1 for the diagnosis of AD. ROC for NRG1 and BACE1 CSF levels in AD patients versus neurological controls

The results of CSF NRG1 and BACE1 levels are shown in Tables 1 and 2 and Fig. 2. For NRG1 ELISA tests, intra- and inter-assay variations were respectively 0.56% and 4.52%; 4.37% of results were under the detection limit, and the median difference of the two tests (normalized for the average) was 0.0093. BACE1 levels were significantly increased in AD and MCI-AD compared to non-AD MCI, other dementias, and controls. No difference was observed between AD and MCI-AD suggesting that the increase of levels is rather early during the evolution of brain AD lesions. Table 2 and Fig. 2 reveal that, after adjustment for age and sex, CSF NRG1 levels were significantly increased in AD and MCI-AD patients as compared to other groups. Further adjustment on the MMSE score and CSF Aβ1–42 levels led to a decrease in the differences between the AD and MCI-AD groups (Table 2, model 2). Surprisingly, no statistically significant correlations were observed in AD and MCI-AD patients between CSF levels of BACE1 and NRG1. Figure 3 shows the discriminatory power of CSF synaptic NRG1 and BACE1 between AD and neurological controls. NRG1 could discriminate AD with an AUC at 0.72 (0.60–0.84). The sensitivity was 72.1%, and the specificity was 67.2%. The discriminatory power of CSF BACE1 between AD and neurological controls has an AUC of 0.78 (0.67–0.89), a sensitivity of 87.2%, and a specificity of 63.6%.

**Table 2:** Relationship between CSF NRG1 and groups of patients, compared to controls. Linear regression analysis models

Groups	CSF NRG1 (log)					
	Unadjusted		Model 1^a^		Model 2^b^	
	*β* (SE)	*p* value	*β* (SE)	*p* value	*β* (SE)	*p* value
Controls	Ref.	–	Ref.	–	Ref.	–
AD	0.30 (0.09)	0.001	0.31 (0.09)	0.001	0.34 (0.11)	0.003
MCI-AD	0.21 (0.11)	0.06	0.22 (0.11)	0.05	0.34 (0.12)	0.004
AD + MCI-AD	0.27 (0.08)	0.001	0.29 (0.09)	0.001	0.34 (0.10)	0.001
Other MCI	0.13 (0.10)	0.18	0.21 (0.10)	0.04	0.24 (0.10)	0.02
Other dementias	0.07 (0.10)	0.46	0.13 (0.11)	0.23	0.11 (0.11)	0.33

^a^Adjusted on age and sex

^b^Model 1 + MMSE score + CSF Aβ1–42

### Correlations between CSF NRG1 1 and cognition

**Fig. 4:**
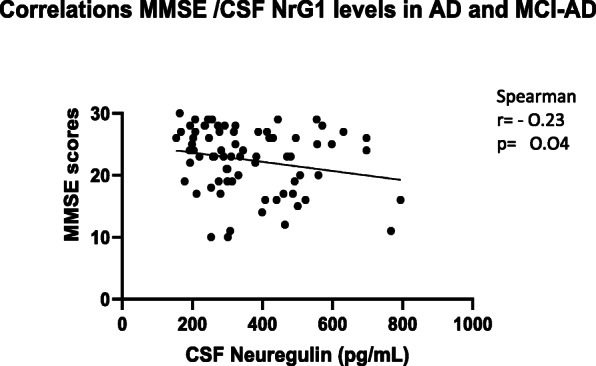
The correlation curve in AD and MCI-AD patients between MMSE scores at intercept and CSF levels of NRG1

**Fig. 5:**
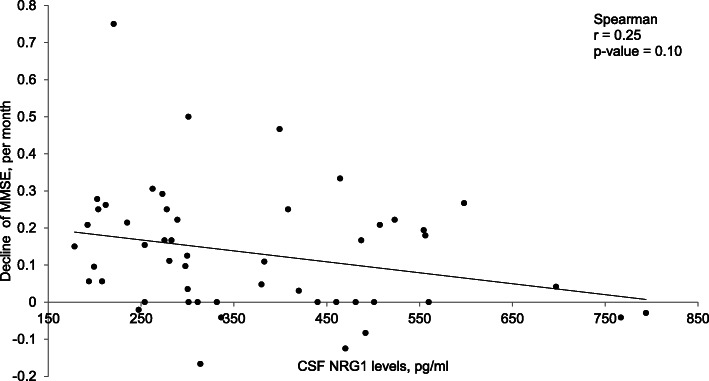
The correlation between the evolutions of MMSE scores per month and the CSF levels of NRG1 in AD patients. In patients with improved MMSE scores, a negative value is depicted

**Fig. 6:**
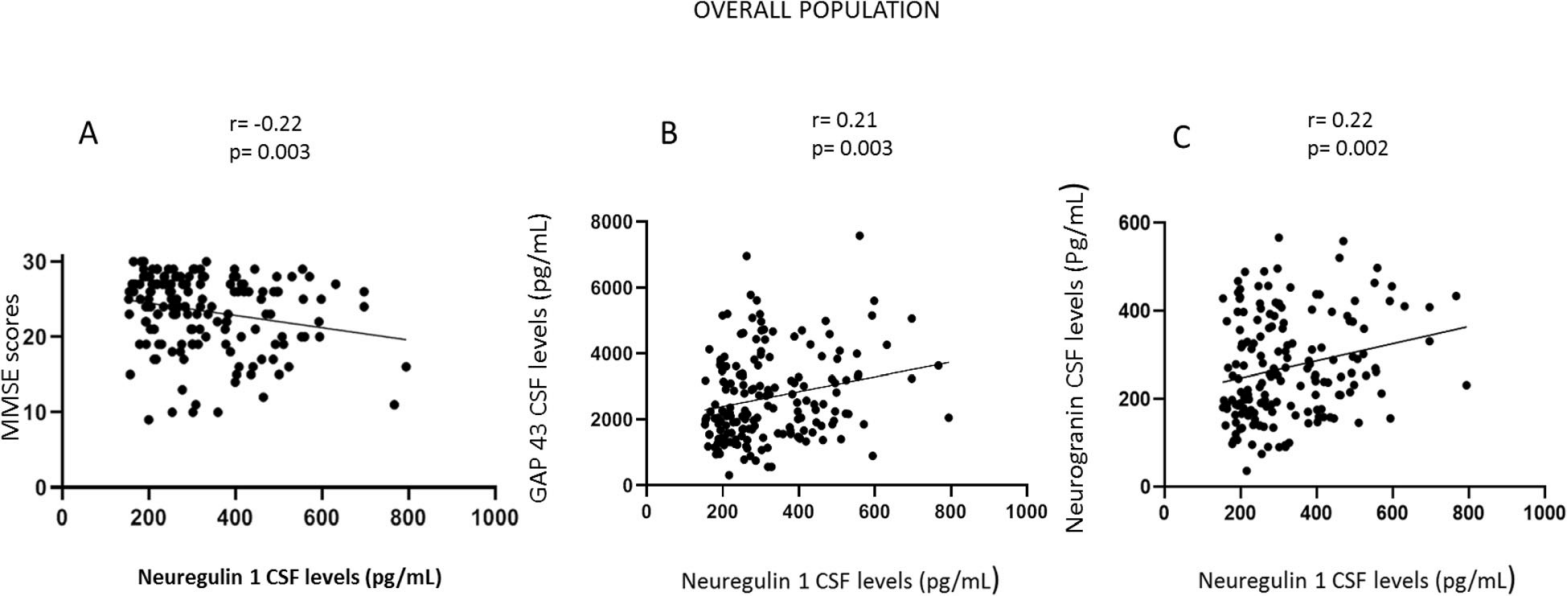
In the overall population, the correlations between CSF NRG1 levels and MMSE scores, GAP 43 levels, and CSF Ng levels

In AD patients, CSF NRG1 levels inversely correlated with MMSE scores (*r* = − 0.44, *p* value = 0.001). This association remained after taking into account multiple testing with Bonferonni correction. Patients with higher MMSE scores had reduced levels of CSF NRG1. This inverse correlation was also present in the group AD and MCI-AD (Fig. 4a) but also in the overall population (Fig. 6a). In addition, AD patients were followed for up to 3 years, and we evaluated the MMSE decline per month for each patient. Figure 5 shows a tendency (*p* = 0.08) for an inverse correlation between the cognitive decline and CSF NRG1 baseline levels in AD patients. Surprisingly, AD patients with low CSF NRG1 levels had a tendency to have a more rapid cognitive decline as compared to patients with higher CSF NRG1 levels.

### Correlations with CSF Aβ, T-tau, P-tau, and synaptic biomarkers

CSF NRG1 levels correlated positively with CSF Aβ1–42 levels in the AD group alone (*p* = 0.03) (Table 3) and in the AD plus MCI-AD group (*p* < 0.001) (Fig. 4b and Supplementary Table 1). After taking into account the Bonferonni correction, CSF NRG1 levels were associated to CSF Aβ1–42 only in the AD plus MCI-AD group. CSF BACE1 levels correlated with Aβ1–40 (*p* < 0.001), T-tau (*p* = 0.002), and P-tau (*p* = 0.004) in AD. No correlation was observed in the AD group between CSF levels of NRG1 and CSF levels of T-tau and P-tau. No correlation was also found in the AD group between CSF levels of BACE1 and Aβ1–42. In the overall population, CSF NRG1 levels positively correlated with CSF GAP 43 levels (*p* < 0.001, Fig. 6b) and with CSF neurogranin levels (*p* < 0.001) (Fig. 6c) suggesting that a substantial part of CSF NRG1 could also originate from the synapses.

**Table 3:** Spearman correlation coefficients between CSF NRG1 and other characteristics in AD patients

	Spearman correlation coefficients (*p* value)							
	CSF neuregulin	Age	MMSE	CSF Aβ1–42	CSF Aβ1–40	CSF tau	CSF P-tau	CSF BACE1
CSF neuregulin	1							
Age	− 0.14 (0.33)	1						
MMSE	− 0.44 (0.001)	0.17 (0.24)	1					
CSF Aβ1–42	0.28 (0.03)	0.01 (0.92)	− 0.24 (0.09)	1				
CSF Aβ1–40	− 0.07 (0.61)	0.13 (0.37)	0.15 (0.32)	0.34 (0.01)	1			
CSF tau	0.06 (0.66)	− 0.10 (0.47)	− 0.11 (0.45)	0.0 (0.98)	0.29 (0.04)	1		
CSF P-tau	− 0.03 (0.81)	− 0.04 (0.78)	− 0.03 (0.83)	0.1 (0.85)	0.32 (0.02)	0.85 (< 0.001)	1	
CSF BACE1	− 0.14 (0.32)	0.08 (0.55)	0.17 (0.23)	0.05 (0.72)	0.67 (< 0.001)	0.41 (0.002)	0.39 (0.004)	1

Correlations between CSF NRG1 levels and other characteristics in AD patients

Spearman correlation coefficient was used for statistical analysis

## Discussion

Our results showed that CSF NRG1 concentrations are enhanced in AD and MCI-AD as compared to controls and other dementias. A correlation was found between CSF NRG1 levels and cognitive status, and a trend with cognitive decline was observed in AD patients. NRG1 could participate in the pathophysiology of AD. The results show increased CSF levels of NRG1 in AD and MCI-AD patients as compared to neurological controls but the discriminatory power of this marker is lower than the usual CSF AD marker such as Aβ1–42, T-tau, and P-tau. Interestingly, CSF NRG1 correlates with Aβ1–42 levels in the AD and MCI-AD groups and correlate with other synaptic biomarkers in the overall population. In AD patients, CSF NRG1 levels negatively correlate with cognition and show a correlation trend with cognitive decline. Synaptic APP and NRG1 are cleaved by BACE1, and further studies will be necessary to explore the beneficial or detrimental effects of increased NRG1 concentrations in AD brains and bring about new pathophysiological information on neurodegeneration in AD. CSF NRG1 levels are already enhanced in MCI-AD patients and modulating the increased activity of synaptic NRG1 in AD might represent an original therapeutic strategy to alter abnormal signaling linked to cognitive decline and neurodegeneration.

NRG1 is a BACE1 substrate, and knowing its various brain physiological functions in neuroprotection, memory modulation, and inflammation, there are several questions that could be addressed.

1. Why are CSF NRG1 levels increased in AD and MCI-AD patients? Increased NRG1 levels could reflect increased BACE1 activity and enhanced synaptic degeneration. It is known that BACE1 is the limiting step of Aβ1–42 production and that BACE1 levels and activity both are increased in AD brains [7]. It is plausible to hypothesize that increased activity of BACE1 may lead to enhanced levels of NRG1 in AD brains and CSF. NRG1 may accumulate in the brain of AD patients [18] as Aβ1–42 but possibly at a much lower level than this peptide, and the major part of this protein could be released into the CSF in AD. This possibility would explain the positive correlation of the two CSF markers although CSF Aβ1–42 levels are reduced in AD whereas CSF NRG1 levels are enhanced in this group. The lack of correlation between CSF levels of NRG1 and BACE1 could be explained by the fact that the precursor of NRG1 can be cleaved by other synaptic proteases than BACE1 and is also regulated in an activity-dependent manner [19].

2. Another question is why are CSF NRG1 levels negatively linked to cognition in AD and MCI-AD groups and in the overall population? Experimental studies have shown that the modulatory effect of NRG1 on cognition has a dose-response curve with an inverted U form [9].. Previous experimental works have revealed that NRG1 can either be beneficial for molecular mechanisms of memory or detrimental for such processes [20, 21]. Low levels of NRG1 at synapses lead to detrimental cognitive effects whereas high levels are also linked to negative cognitive effects. The positive effect of NRG1 on cognition is limited to a medium range of concentrations. One could propose that in cognitively normal individuals, medium levels of NRG1 are cleaved by BACE1 and contact ErRb4 post-synaptic receptors without increased release in the CSF. When AD brain lesions appear, increased levels of NRG1 may temporarily compensate Aβ neurotoxicity at the synapse, acting as a trophic support and a cognitive enhancing factor. This trophic support and neuroprotective action may or may not persist in more advanced AD cases with relatively increased CSF NRG1 levels as compared to patients with low CSF NRG1 concentrations, but detrimental effects on cognition could also be associated with high NRG1 levels. Further studies in a larger cohort of patients and controls will be needed to confirm these possibilities. In addition, it will be interesting to study in the future if a difference can be found between the memory action and the neuroprotective action of NRG1 depending upon efficient synaptic levels. Previous reports also revealed that the couple NRG1/ErB4 receptors can also induce inflammation and the release of cytokines that may have positive effects as well as neurotoxic consequences [22–24]. Further studies will be needed to evaluate if CSF NRG1 levels in AD are correlated with CSF inflammatory markers and cytokines.

3. Another question that could be addressed is how our results interfere with the putative role of NRG1 in the regulation of memory and neuroinflammation. Neuregulin gene polymorphisms in humans are associated with schizophrenia [25] marked by the presence of cognitive deficits. The couple NRG1/ErbB4 receptors is linked to trophic properties. NRG1 action is modulated by neuregulin receptor degradation protein 1 (Nrdp1) which is able to induce the suppression of the ErbB4 receptor [26]. The levels of this neurotoxic E3 ubiquitin-protein ligase (Ndrp1) are increased by LPS-induced neuroinflammation [27] which is also under the control of the eukaryotic translation initiation factor 2-alpha kinase 2 (PKR) [28] whose levels are enhanced in AD brains and CSF [29, 30]. The reason why CSF NRG1 levels tend to be negatively correlated with cognitive decline in AD patients is not known. This result could be linked to a variation of the NRG1/ErbB4 trophic properties in clinically more advanced AD patients because of the inverted U shape of the dose/response curve. Further studies will be needed to decipher the interactive modulatory effects of NRG1, ErbB4, Nrdp1, PKR, and inflammation on cognition in AD.

Finally, recent clinical results assessing the BACE inhibitor verubecestat have revealed a cognitive worsening in patients with prodromal AD [31]. Patients at this stage of the disease may be more sensitive than more advanced AD patients to the detrimental synaptic effects of BACE1 inhibition [32–34]. Whether or not these cognitive effects are linked to a modulation of NRG1 release induced by the BACE1 inhibitor remains to be explored.

This study has some limitations. First, new evaluations in a larger cohort of patients and controls are needed to establish if CSF NRG1 could be increased in AD and MCI-AD. Furthermore, the ability of CSF NRG1 to discriminate AD from controls, as assessed by the ROC curve analysis, was modest. Its dosage allows a better understanding of the dynamic between BACE1 and β-amyloid peptides levels in the CSF but prevents to use it as a discriminant biomarker of the disease in a clinical setting. Second, more information about CSF concentrations of the couple NRG1/erbB4 receptors would be necessary to obtain, because most synaptic NRG1 actions are made via this pathway [35, 36], and a previous study has shown that brain ErbB4 levels could be involved in the progression of AD [37]. Third, the evaluation of CSF NRG1 in cognitively normal individuals at risk for AD (positive amyloid PET for example) would be interesting to determine and to correlate with neurodegenerative markers and cognitive evolutions. Fourth, the evaluation of serum and CSF levels of NRG1 may be useful in the future to determine if blood and CSF levels are correlated [38].

## Conclusions

CSF levels of the BACE1 substrate NRG1 are modified in AD and MCI-AD patients. Knowing that NRG1 is implicated in neuroprotection, memory modulation, and neuroinflammation, this protein could represent a new CSF marker reflecting compensatory mechanisms in AD. Targeting brain NRG1 activity and synaptic NRG1 pathway in MCI-AD and AD patients could be a possible new way to attenuate cognitive decline and neuronal demise.

## Supplementary information

**Additional file 1: Supplementary Table 1.** Spearman correlation coefficients between CSF NRG1 and characteristics in AD + MCI-AD patients.

## Data Availability

All data generated or analyzed during this study are included in this published article.

## Abbreviations

BACE1: Beta-site amyloid precursor protein cleaving enzyme 1
NRG1: Neuregulin1
MCI: Mild cognitive impairment
AD: Alzheimer’s disease
CSF: Cerebrospinal fluid
erbB4: Receptor tyrosine-protein kinase erbB-4
PKR: Eukaryotic translation initiation factor 2-alpha kinase 2
Nrdp1: Neuregulin receptor degradation protein 1
AUC: Area under the curve
APP: Amyloid precursor protein
ADAM: Disintegrin and metalloproteinase
PET: Positron emission tomography
GAP 43: Growth-associated protein 43
Ng: Neurogranin
T-tau: Total tau
P-tau: Phosphorylated tau
