# Supplementary material for: CSF levels of the BACE1 substrate NRG1 correlate with cognition in Alzheimer’s disease
Source: Alzheimers Res Ther. 2020 Jul 20;12:88. doi: 10.1186/s13195-020-00655-w (PMC7372801; doi:10.1186/s13195-020-00655-w)
Supplement: Supplementary file 1 — Additional file 1: Supplementary Table 1. Spearman correlation coefficients between CSF NRG1 and characteristics in AD + MCI-AD patients. [file 13195_2020_655_MOESM1_ESM.docx]

**Supplementary Table 1. Spearman correlation coefficients between CSF NRG1 and characteristics in AD + MCI-AD patients**

|  |  |  |  |  |  |  |  |  |
| --- | --- | --- | --- | --- | --- | --- | --- | --- |
|  | CSF Neuregulin | Age | MMSE | CSF Aβ42 | CSF Aβ40 | CSF Tau | CSF p-Tau 181 | CSF Bace-1 |
| CSF Neuregulin | 1 |  |  |  |  |  |  |  |
| Age | -0.01 (0.95) | 1 |  |  |  |  |  |  |
| MMSE | -0.33 (0.005) | 0.14 (0.24) | 1 |  |  |  |  |  |
| CSF Aβ42 | 0.23 (0.045) | 0.02 (0.85) | 0.02 (0.89) | 1 |  |  |  |  |
| CSF Aβ40 | 0.06 (0.63) | 0.11 (0.37) | 0.01 (0.99) | 0.40 (<0.001) | 1 |  |  |  |
| CSF Tau | 0.10 (0.39) | -0.14 (0.24) | -0.28 (0.017) | 0.01 (0.98) | 0.32 (0.007) | 1 |  |  |
| CSF p-Tau 181 | -0.04 (0.74) | 0.01 (0.92) | -0.21 (0.07) | -0.01 (0.86) | 0.32 (0.008) | 0.84 (<0.001) | 1 |  |
| CSF Bace-1 | -0.056 (0.64) | 0.10 (0.40) | 0.01 (0.91) | 0.18 (0.13) | 0.69 (<0.001) | 0.42 (<0.001) | 0.42 (<0.001) | 1 |
|  |  |  |  |  |  |  |  |  |
